# Running the Stop Sign: Readthrough of a Premature UAG Termination Signal in the Translation of a Zebrafish (*Danio rerio*) Taurine Biosynthetic Enzyme

**DOI:** 10.3390/md15060162

**Published:** 2017-06-03

**Authors:** Mary E. M. Larkin, Allen R. Place

**Affiliations:** Institute of Marine and Environmental Technology, 701 E. Pratt Street, Baltimore, MD 21202, USA

**Keywords:** translational readthrough, taurine, CSAD, zebrafish, *Danio rerio*

## Abstract

The UAG termination codon is generally recognized as the least efficient and least frequently used of the three universal stop codons. This is substantiated by numerous studies in an array of organisms. We present here evidence of a translational readthrough of a mutant nonsense UAG codon in the transcript from the cysteine sulfinic acid decarboxylase (*csad*) gene (ENSDARG00000026348) in zebrafish. The *csad* gene encodes the terminal enzyme in the taurine biosynthetic pathway. Taurine is a critical amino acid for all animals, playing several essential roles throughout the body, including modulation of the immune system. The sa9430 zebrafish strain (ZDB-ALT-130411-5055) has a point mutation leading to a premature stop codon (UAG) 20 amino acids 5’ of the normal stop codon, UGA. Data from immunoblotting, enzyme activity assays, and mass spectrometry provide evidence that the mutant is making a CSAD protein identical to that of the wild-type (XP_009295318.1) in terms of size, activity, and amino acid sequence. UAG readthrough has been described in several species, but this is the first presentation of a case in fish. Also presented are the first data substantiating the ability of a fish CSAD to utilize cysteic acid, an alternative to the standard substrate cysteine sulfinic acid, to produce taurine.

## 1. Introduction

**Figure 1:**
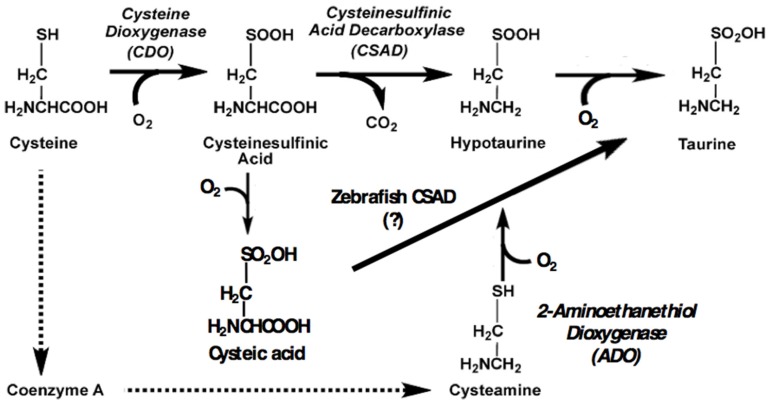
Cysteine sulfinic acid decarboxylase (CSAD) is the terminal enzyme in the taurine biosynthetic pathway. (Modified from Vitvitsky et al [18]).

To assess a possible amelioratory role for taurine in dietary induced inflammation, our laboratory seeks to procure a strain of zebrafish deficient in endogenous taurine synthesis. This will enable clear observation of the effects of graded dietary supplementation. Taurine (2-aminoethanesulfonic acid) is a critical amino acid for animals and must be synthesized de novo or obtained through the diet. For taurine synthesizers, the cysteine sulfinic acid decarboxylase (CSAD) enzyme (XP_009295318.1) catalyzes the terminal reaction in the primary biosynthetic pathway (Figure 1). Essential roles for taurine include osmoregulation, bile salt conjugation, and protection from oxidative stress [1]. Species lacking sufficient endogenous synthesis require dietary supplementation. This is particularly apparent in carnivores who tend to have low levels of CSAD or none at all. Strictly carnivorous cats have low levels of CSAD but require high levels of taurine as well as methionine, which along with cysteine is a precursor of taurine synthesis [2,3]. In fish, deficiencies in taurine production have become increasingly apparent as aquaculture feeds exchange greater proportions of fish meal for plant protein sources containing no taurine. The supplementation requirements of several commercially relevant species have been described, including by our laboratory for the strict marine carnivore, cobia (*Rachycentron canadum*). Taurine synthesizers also benefit in terms of growth from supplementation in the feed, with 1.5% being the standard amount in commercial formulations. [4,5,6,7]. The U.S. FDA (United States Food and Drug Administration) recently approved taurine supplementation of feed for farmed fish intended for human consumption [8].

Knocking out the *csad* gene in mice leads to an 83% reduction in plasma levels of taurine, with resulting mortality within 24 h of birth by the third generation of inbred homozygotes [9]. Residual production of taurine may be via the cysteamine pathway (Figure 1). Cats, as insufficient synthesizers of taurine, exhibit retinal degeneration and hepatic lipidosis when fed a diet without taurine supplementation [2,3]. Taurine-deficient fish display similar liver maladies, largely expected since fish conjugate bile salts to taurine [5,6]. CSAD mRNA is found in the earliest stages of zebrafish embryonic development, including in the yolk at the gastrula stage, and in both the yolk and notochord in the somite and pharyngula stages. In the pharygula stage, 24–48 hpf (hours post fertilization), CSAD expression is evident in several additional tissues including the liver, brain, and optic cup [10,11]. Knockdown of CSAD mRNA or TauT (taurine transporter) mRNA during early embryonic development results in cardiomyopathy and mortality in zebrafish [11,12]. Taurine is also an immunomodulator, mitigating inflammation by scavenging free radicals and inhibiting the production of several potent inflammatory agents including tumor necrosis factor-α (TNF-α), interleukins, prostaglandins, superoxide anion, and nitric oxide [13,14].

The sa9430 mutant zebrafish strain (Sanger, Zebrafish Mutation Project, [15]) was acquired as a potential knockout for *csad* suitable for our dietary studies. This strain has a C→T point mutation in the single copy of the *csad* gene on chromosome 23. The mutation alters a codon that would normally be translated to glutamine to a UAG stop codon 20 amino acids prior to the normal UGA stop codon. No phenotype has been described for this mutant, but commercial feeds typically contain adequate dietary taurine and the lack of de novo synthesis may be masked. Zebrafish deficient in taurine synthesis that do not receive dietary supplementation are expected to exhibit pathologies similar to those seen in the knockdown studies, including mortality. Since our fish were reared and maintained on a commercial taurine-supplemented diet, as well as taurine-containing live artemia, gross changes in phenotype were not necessarily expected. However, we did not anticipate the results presented here from immunoblotting, enzyme activity assays, and mass spectrometry which show that the sa9430 mutant is producing wild-type CSAD protein.

The CSAD of some animals can utilize cysteic acid as a substrate for taurine synthesis, including cats and rats [16,17]. In this pathway, there is the direct conversion of cysteic acid to taurine without a hypotaurine intermediate. Data presented here reveal the ability of zebrafish CSAD to utilize cysteic acid as an alternative to cysteine sulfinic acid as a substrate for taurine synthesis.

## 2. Results

### 2.1. Standard Feeds for Zebrafish Contain Taurine

At 5–10 dpf (days post fertilization) larvae are fed paramecia until their mouths are sufficiently large to consume artemia or the smallest commercial pelleted feeds. Our analysis by HPLC detects no taurine in paramecia, so during this feeding period, the larvae are dependent upon maternal contribution of taurine and *csad* mRNA as well as their own de novo synthesis [11]. At approximately 10 dpf, zebrafish begin consuming live artemia, which are grown without taurine enrichment in our facility. According to LC-MS analysis, artemia naturally contain 0.033% ± 0.003% taurine. At this stage of development, zebrafish larvae can also begin consuming the smallest pelleted feeds. These formulations, commonly fed to zebrafish in our facility, were found to contain approximately 1.5% taurine. 

### 2.2. Zebrafish Synthesize Sufficient Taurine for Homeostasis

Juvenile zebrafish were fed diets containing either zero or 4% taurine for 8 weeks. Survival and growth for zebrafish on both diets were equivalent. Whole body taurine values at the conclusion of the feeding trial were 1.37 ± 0.03% for fish on the zero taurine diet and 2.04 ± 0.28% for fish on the 4% taurine diet. These differences are statistically significant. Expression of taurine biosynthetic genes CSAD, CDO (cysteine dioxygenase), and ADO (2-aminoethanthiol dioxygenase), as well as TauT, were confirmed by RT-PCR [19].

### 2.3. Wild-type and sa9430 Fish Have Similar Fitness Despite Lower Taurine Levels in sa9430 Embryos

There were no apparent differences in survivorship or fecundity between the wild-type and sa9430 strains. Wild-type embryos at ~1 hpf contain 10 pmol, taurine, and sa9430 embryos contain 4 pmol taurine. Both 5-week-old wild-type and sa9430 juveniles consuming taurine-supplemented feed for 3.5 weeks had approximately 2.7 µmol taurine per fish. 

### 2.4. Wild-type and sa9430 CSAD Proteins Have Identical Modeled Conformations

**Figure 2:**
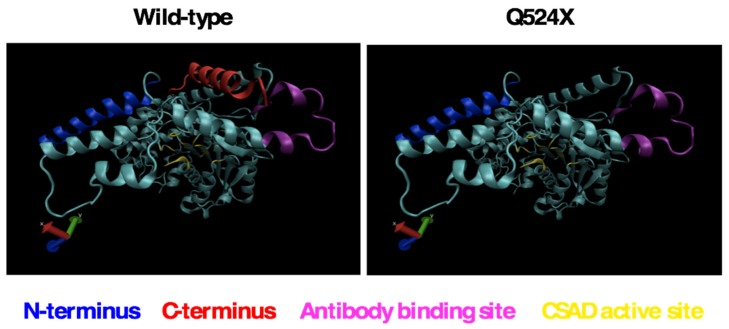
Modeling of the sa9430 mutant protein suggests the possibility of a fully functional active site even in a truncated protein. Modeling was performed using the Phyre2 program as described in Materials and Methods [20].

Computer modeling of the wild-type and sa9430 CSAD proteins suggest that a mutant protein, if properly folded and not degraded, may have a functional active site even if truncated (Figure 2). Specific amino acids in the ligand binding site are also predicted to be the same in the wild-type and mutant CSAD (data not shown).

### 2.5. Nucleotides That Border the UAG May Increase Readthrough Frequency

**Figure 3:**
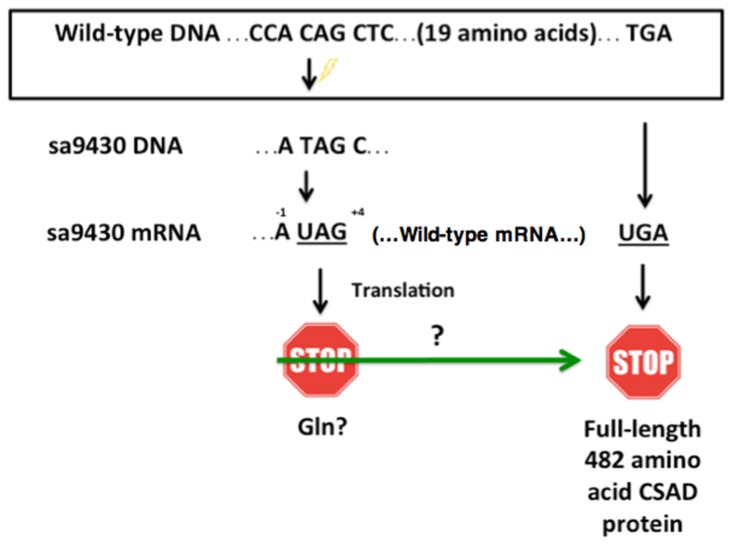
The sa9430 zebrafish strain has a premature UAG stop codon with adjacent nucleotides that may increase probability of a readthrough. The sa9430 zebrafish strain has a C→T point mutation that changes the codon CAG (codes for glutamine) to UAG (translational stop signal). The presence of an adenine residue −1 of the UAG and a cytosine +4 of the UAG may contribute to a readthrough of the stop codon. If readthrough occurs, translation should terminate at the wild-type UGA stop signal 20 codons downstream.

The sa9430 zebrafish strain has an adenine in the −1 position and a pyrimidine in the +4 position relative to the nonsense UAG stop codon (Figure 3). Sequence information supplied with the strain was confirmed by PCR amplification and sequencing of a 364-bp (base pair) region of DNA containing the C→T point mutation. If the same trends hold true for zebrafish as for other studied organisms, these particular nucleotides flanking the UAG could increase frequency of readthrough [21,22]. UAG can be translated as glutamine during readthrough, which would result in a wild-type protein [23,24,25,26].

### 2.6. sa9430 CSAD Is the Same Size as the Wild-Type CSAD

**Figure 4:**
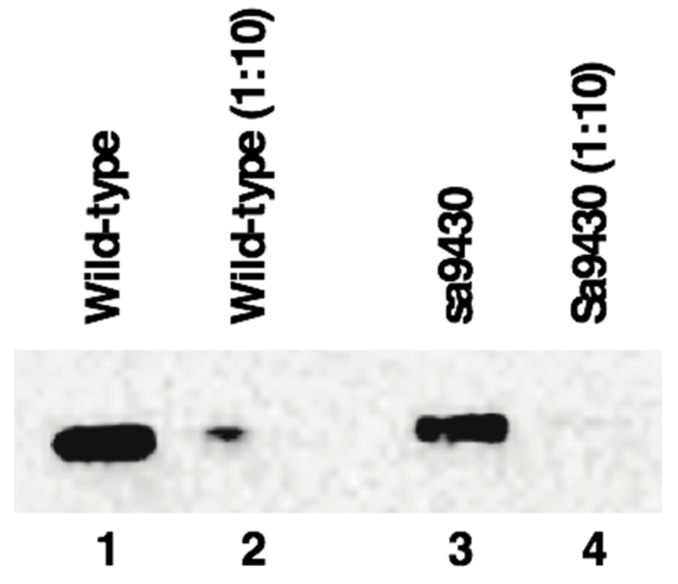
Wild-type CSAD and sa9430 CSAD are an estimated 58.1 kDa and 60.0 kDa, respectively, using molecular weight standards for reference for analysis using the Image Lab software (Bio-Rad). These are likely equivalent values within the error range of the program. Liver proteins were extracted and immunoblotting performed as described in Materials and Methods. Lanes 1–4 contain 3.7 μg, 0.37 μg, 2.6 μg, and 0.26 μg protein, respectively. Increasing the run time in the gel did not reveal a second band indicating production of a truncated CSAD by sa9430 in addition to the wild-type.

Liver proteins from wild-type and sa9430 zebrafish were extracted, genotypes confirmed, and immunoblotting performed. For genotyping, a 364-base region was sequenced which includes the site of the C→T point mutation in the sa9430 mutant. As shown in Figure 4, protein sizes are the same for detectable CSAD from both wild-type and sa9430. Immunoblotting of heterozygotes resulted in a single band of the same size (data not shown). The binding site for the anti-CSAD antibody is predicted to be present in a truncated CSAD in addition to the wild-type as shown in the protein models in Figure 2.

Though the gradient gel type used is sufficient to visualize a size difference of 20 amino acids (2.5 kDa), we confirmed the absence of a smaller band by running the gel 30% longer (data not shown). In addition, to be sure a concentrated level of protein was not masking a smaller band, the 1:10 dilutions for each strain were analyzed (lanes 2 and 4 of Figure 1). The absence of a visible truncated protein suggests that if any is being produced it is below detectable levels or being degraded.

### 2.7. Wild-Type and sa9430 CSAD Enzymes Catalyze the Conversion of Cysteic Acid to Taurine at Comparable Rates

For analysis of enzyme activity, liver proteins were extracted from wild-type and F2 generation sa9430 homozygotes. Based on computer modeling suggesting that the active site would be preserved even in a truncated protein and immunoblotting data indicating a full-length protein in the sa9430 mutant, we anticipated some level of functional enzymatic activity. As shown in Table 1, the wild-type and sa9430 CSAD convert cysteic acid to taurine at statistically indistinguishable rates.

### 2.8. Mass Spectrometry of Tryptic Peptides Indicates sa9430 Is Producing A Wild-Type CSAD Protein

**Figure 5:**
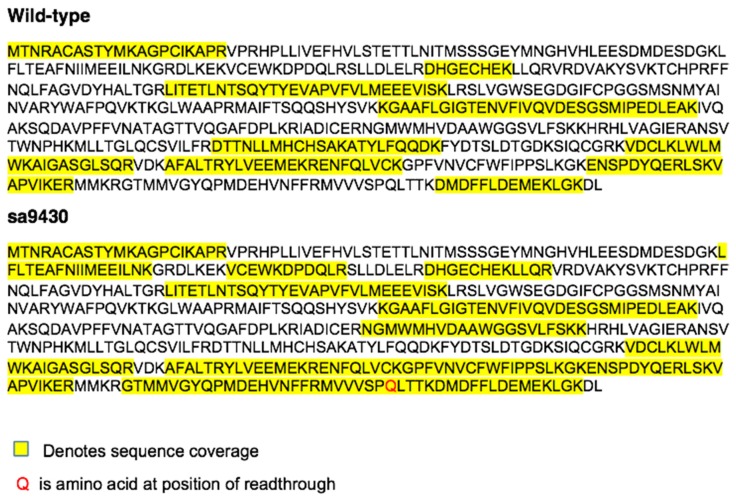
The sa9430 zebrafish strain is producing a full-length CSAD protein with glutamine at the site of readthrough, the same amino acid as in the wild-type. Sequence coverage is 542 amino acids (62 kDa), which corresponds to the CSAD X1 isoform (XP_009295318.1). Proteomics analysis was performed as described in Materials and Methods.

Total protein from liver extracts was immunopurified using an anti-CSAD antibody and prepared for mass spectrometry (MS). Peptides detected by tandem MS were aligned to the published CSAD sequence 482 amino acids in length (NP_001007349) as well as the predicted X1 isoform 544 amino acids in length (XP_009295318.1). The sa9430 zebrafish strain is producing full-length CSAD protein, with sequence coverage of 542 amino acids for both wild-type and sa9430 (Figure 5). The nonsense UAG in sa9430 is being translated to glutamine, the same amino acid as in the corresponding position in the wild-type. N-terminal sequence coverage matches the predicted X1 isoform.

## 3. Discussion

In mammals, the UAG codon has been described as notoriously “leaky”, and the nucleotides preceding and following the stop codon influence the frequency of readthrough. Readthrough frequency is increased in mammals when an adenine is in the −1 position, the nucleotide preceding the UAG stop codon. A pyrimidine in the +4 position, which corresponds to the nucleotide directly following the UAG, is associated with a greater chance of readthrough as compared to a purine [21]. The sa9430 zebrafish strain has both an adenine in the −1 position and a pyrimidine (cytosine) in the +4 position. 

Stop codon efficiency and frequency are correlated. In many organisms, UAG is not only the least efficient in terminating translation, it is also the least frequently occurring codon [27]. In lower eukaryotes such as *Candida* and *Drosophila*, UAGA is the most efficient and frequent sequence, while UAGC is the least efficient and frequent sequence [22]. UAGC is the sequence found in the sa9430 zebrafish strain (Figure 3). A study on stop codon frequency in blunt snout bream (*Megalobrama amblycephala*) reported that UAG has a relative synonymous codon usage (RSCU) value of 0.59 as compared to 1.11 for UAA and 1.29 for UGA [28]. RCSU value is a measure of codon bias, and the score of <1 for UAG signifies its less frequent use as compared to the other codons [29]. It may also be less efficient at terminating translation.

Translational readthrough is mediated by two predominant mechanisms. One is through the action of suppressor tRNAs, which incorporate an amino acid at the site of the stop codon. The other is through the pairing of a near-cognate tRNA with the stop codon. In *S. cerevisiae*, suppressor tRNA(Gln) have been identified that insert glutamine at UAG stop codons. Tyrosine or lysine can also be inserted [25]. A study of virally induced termination suppression also demonstrated UAG translation to glutamine [26]. These same three amino acids have been shown to be inserted at a UAG stop codon as a result of near-cognate tRNA pairing. This is accomplished by mispairing at position 1 as well as at the classic wobble position 3 of the nonsense codon [23,24]. In this case, it is probable that mispairing of a tRNA complementary to CAG is occurring at position 1, resulting in the incorporation of a glutamine at UAG. 

Taurine has been shown to be incorporated into modified uridines in mitochondrial tRNAs of sea squirts, cows, and humans. Lack of this taurine incorporation at a wobble position uridine results in weak codon-anticodon pairing and is associated with defective translation and subsequent disease [30,31]. It is challenging to speculate as to precisely which mechanisms are orchestrating the readthrough. The lower levels of taurine in the sa9430 embryos as compared to the wild-type may have to do with the pool of tRNAs present early in development. 

It is notable that activity assays using the standard substrate, cysteine sulfinic acid, did not yield conclusive results (data not shown). Substrate levels reduced over the course of the assay without a resulting increase in taurine levels. Another enzyme present in the liver may compete for this substrate.

It was important to analyze F2 homozygotes in immunoblotting and activity assays. Maternal influence on offspring phenotype can be particularly confounding in the first generation. This was apparent in the studies of CSAD knockout in mice, with deleterious effects increasing over generations [9]. Analyzing second generation homozygotes strengthens the proposal that mutants are making wild-type protein and there is no change in phenotype.

This work establishes why no aberrant phenotype has been previously described for the sa9430 strain. Despite the altered *csad* genotype, there is expression of a wild-type CSAD protein. Since this strain is unsuitable for studying the effects of dietary taurine supplementation in a taurine-deficient fish, our laboratory is in the process of using CRISPR/Cas9 technology to generate a *csad* knockout. Assessing the capacity for taurine to alleviate inflammation has implications beyond zebrafish. Findings will be useful in the development of plant-based feeds for commercially relevant fish, with taurine potentially mitigating the inflammatory potential of plant ingredients which are otherwise desirable in terms of supply and cost. This research may also lead to improvement in treatments for IBD (inflammatory bowel disease). In a zebrafish model of IBD, administration of taurine mollified chemically-induced degeneration of the intestinal mucosa and other symptoms of inflammatory bowel disease [13,32].

## 4. Materials and Methods

### 4.1. Computer Modeling of CSAD

Wild-type (ENSDARG00000026348) and sa9430 (ZBD-ALT-130411-5055) CSAD proteins were modeled using the Phyre2 web portal [20]. Sequences were aligned to models of the human CSAD protein. 3D LigandSite was used to predict the ligand binding site amino acids for both wild-type and sa9430 [33].

### 4.2. Zebrafish Strains and Maintenance

Wild-type and sa9430 zebrafish were maintained at the zebrafish facility of the Aquaculture Research Center at the Institute of Marine and Environmental Technology (Institutional Animal Care and Use Committee at University of Maryland, Baltimore #0315011, approved April 2015). Fish were maintained on a 14 h light, 10 h dark cycle at 28.5 °C. Larvae were fed a combination of paramecia, artemia, and GEMMA Micro 75 (Skretting). At the appropriate size, fish transitioned to GEMMA Micro 150, then 300, with occasional additions of artemia, particularly in the week preceding mating. Heterozygous embryos of *csad* sa9430 Tb (Sanger, Zebrafish Mutation Project) were raised and naturally bred to obtain homozygotes with wild-type *csad* and homozygotes with sa9430 mutant *csad*.

### 4.3. Genotyping

For confirmation of genotype, caudal fin clips were obtained and immersed in genomic DNA extraction buffer (50 mM KCl, 10 mM Tris-HCl (pH = 8.0), 150 mM MgCl_2_, 0.3% Tween-20, and 0.3% NP40 in sterile MilliQ water), boiled at 95–100 °C for 15 min, cooled on ice, and digested with proteinase K at 55 °C for 1–3 h. After digestion, lysates were boiled at 95–100 °C for 15 min to inactivate proteinase K and centrifuged for 3 min at 12,000× *g* [34]. The resulting genomic DNA was amplified by PCR using zebrafish *csad*-specific primers:

Forward: 5’ ACGTGGCGCCAGTCATTAAA 3’
Reverse: 5’ GATGCCAATCGTTTGACCAGT 3’

Sequencing of the 364-base-pair PCR product was performed in a 10 µL reaction volume consisting of 40–150 ng PCR product, 3 pmol of primer, 0.5 µL Big Dye v3.1 sequencing mix and 1.5 µL 5X sequencing buffer (Applied Biosystems, ThermoFisher Scientific, Waltham, MA, USA). Cycling parameters were 95 °C for 5 min, followed by 50 cycles at 95 °C for 15 s, 50 °C for 15 s, 60 °C for 4 min. The sequencing product was purified by adding 60 µL 100% isopropanol and 30 µL H_2_O, mixing thoroughly, incubating at room temperature for 30 min, and centrifuging at 2000× *g* for 30 min. The supernatant was decanted and 100 µL 70% isopropanol was added to wash the DNA, followed by another centrifugation at 2000× *g* for 14 min and decanting of the supernatant. Labeled products were air dried for 20–30 min before addition of 10 µL HI-DI formamide from Applied Biosystems. The mixture was heated at 95 °C for 2 min and then immediately put on ice. The denatured product was sequenced using an Applied Biosystems 3130XL Genetic Analyzer (ThermoFisher Scientific, Waltham, MA, USA) and compared with the published sequences for the wild-type (ENSDARG00000026348) and sa9430 (ZBD-ALT-130411-5055) strains using the Sequencher program (Version 5.0.1, Gene Codes, Ann Arbor, MI, USA).

### 4.4. Zebrafish Feeding Trials with and Without Supplemental Taurine

This work was performed by Aaron Watson, PhD. Descriptions of the feeding trial, sample preparation, and RT-PCR can be found in his thesis [19], and LC-MS methods to obtain taurine values were performed as described in a publication on taurine leaching [35]. 

### 4.5. Liver Protein Isolation

Zebrafish were euthanized by rapid cooling followed by decapitation. Livers were isolated from wild-type and sa9430 zebrafish and frozen at −80 °C. Liver tissue was homogenized in buffer containing 60 mM Potassium phosphate (pH 7.4), 5 mM DTT, 50 mM sucrose, and 0.5 µM pyridoxal-5’–phosphate (PLP) at a volume of approximately 1:15 weight (mg): lysis buffer volume (μL) [36]. Homogenization was achieved by vortexing and pipetting up and down with a P200 micropipettor. Samples were centrifuged for 5 min at 1500× *g* and supernatant containing the protein fraction collected. For the activity assay, the supernatant was dialyzed in a Slide-a-Lyzer Mini Dialysis Unit (ThermoFisher Scientific, Waltham, MA, USA) for 2 h at 4 °C in the homogenization buffer according to manufacturer’s instructions. Total protein concentration was determined using the Qubit Assay (ThermoFisher Scientific, Waltham, MA, USA).

### 4.6. Immunoblotting

Protein extracts were prepared as described in Section 4.5, combined with standard SDS-PAGE sample buffer, heated for 3 minutes at 95 degrees C, and centrifuged for one minute at 10,000× *g*. For the immunoblot shown in Figure 4, lanes 1–4 of a 4%–12% Bis-Tris protein gel (NuPAGE Novex, (ThermoFisher Scientific, Waltham, MA, USA)) were loaded with 3.7 μg, 0.37 μg, 2.6 μg, and 0.26 μg protein, respectively, and run according to the manufacturer’s protocol for 32 min using MOPS running buffer. Proteins were transferred to a PVDF membrane in the Trans-Blot Turbo Transfer System (Bio-Rad, Hercules, CA, USA). Immunoblotting was performed in the iBind Western System (ThermoFisher Scientific, Waltham, MA, USA) using rabbit anti-zebrafish CSAD antibody at a dilution of 1:1000 (#6862, provided by Plant Sensory Systems, LLC, Halethorpe, MD, USA), as the primary antibody, and goat anti-rabbit IgG H&L HRP conjugate at a dilution of 1:2000 (Bio-Rad, Hercules, CA, USA) as the secondary antibody. A chemiluminescent signal was generated with addition of Clarity Western ECL substrate and imaged in a ChemiDoc Touch Imaging System (Bio-Rad, Hercules, CA, USA).

### 4.7. CSAD Activity Assay

For the activity assay, reaction mixtures were prepared containing 85 μL buffer (same as the homogenization buffer in Section 4.5) and 5 μL total protein from liver (4.4 μg wild-type or 7.1 μg sa9430, Section 4.5). The CSAD enzyme and its PLP cofactor in the buffer were allowed to incubate at 23 °C for 15 min before proceeding with the assay at the same temperature. The time points for the assay were 0, 30, and 90 min. Beginning with the 90-min time point, 10 μL 50 mM cysteic acid (substrate for CSAD) was added to the reaction mixtures. Immediately following the addition of the substrate at the 0-time point, all reactions were stopped with addition of 100 μL (volume equal to total reaction volume) ice cold ethanol with 5% acetic acid. Reaction mixtures were centrifuged at 350× *g* for 10 min 100 μL supernatant was transferred to a clean microcentrifuge tube and dried by evaporation at 70 °C. 

### 4.8. Amino Acid Analysis by HPLC

Dry samples were suspended in 0.1N HCl and filtered through 0.45 micron filters (EMD Millipore, Billerica, MA, USA). 5 μl of the filtered extracts was derivatized according to the AccQTag Ultra Derivitization Kit protocol (Waters Corporation, Milford, MA, USA). Amino acids were analyzed using an Agilent 1260 Infinity High Performance Liquid Chromatography System equipped with ChemStation (Agilent Technologies, Santa Clara, CA, USA) by injecting 5 μL of the derivatization mix onto an AccQTag Amino Acid Analysis C18 (Waters, Milford, MA, USA) 4.0 μm 3.9 × 150 mm column heated at 37 °C. Amino acids were eluted at 1.0 mL·min^−1^ flow with a mix of 10-fold diluted AccQTag Ultra Eluent (C; Waters Corporation, Milford, MA, USA), ultra-pure water (A) and acetonitrile (B) according to the following gradient: initial, 98.0% C/2.0% B; 2.0 min, 97.5% C/2.5% B; 25.0 min, 95.0% C/5.0% B; 30.5 min, 94.9% C/5.1% B; 33.0 min, 91.0% C/9.0% B; 38 min, 40.0% A/60.0% B; 43 min, 98.0% C/2.0% B. Derivatized amino acids were detected at 260 nm using a photo diode array detector. Amount of amino acids was expressed in g per g of dry weight of sample (% DW) making reference to AABA signal, external calibration curve of standard hydrolysate amino acids and dry weight of samples. 

### 4.9. Preparation of Embryos for Amino Acid Analysis by HPLC

Fifty 1-hpf F2 embryos were collected from each of two matings of wild-type fish as well as 50 from two matings of homozygous sa9430 fish. Lyophilized embryos were extracted in 70% ethanol containing 0.154 mM d-norleucine for evaluation of extraction efficiency. Samples were sonicated for 60 min at 25 °C in a bath sonicator (Branson 1200, Emerson, Danbury, CT, USA) followed by centrifugation at 350× *g* for 10 min (IEC, ThermoFisher Scientific, Waltham, MA, USA). The ethanol fraction was retained and dried. These samples were then analyzed by HPLC as described in Section 4.8.

### 4.10. Preparation of Juveniles for Amino Acid Analysis by HPLC

Six 5-week-old F1 juveniles (2 wild-type, 2 heterozygotes, and 2 sa9430 mutants) were homogenized by bead beating for 30 s at 4 m/s in 300 μL cold PBS with Pierce protease inhibitors (Thermofisher Scientific, Waltham, MA, USA). Samples were centrifuged for 5 min at 1500× *g* at 4 °C. Supernatant was filtered through an Amicon Ultra 3K Device (EMD Millipore, Billerica, MA, USA), dried, and then analyzed by HPLC as described in Section 4.8.

### 4.11. Preparation of Paramecia and Artemia for Taurine Analysis

Samples of paramecia and artemia used to feed zebrafish in our facility were centrifuged at 350× *g* for 10 minutes (IEC, ThermoFisher Scientific, Waltham, MA, USA), and the pellet was retained and lyophilized. Samples were resuspended in 70% methanol and sonicated for 60 min at 25 °C in a bath sonicator (Branson 1200). Following centrifugation at 350× *g* for 10 min (IEC), the methanol fraction was retained and dried. These samples were then prepared for taurine analysis by HPLC (Section 4.8) or LC-MS [35], respectively. Taurine levels in artemia were analyzed by Aaron Watson, Ph.D*.*

### 4.12. Preparation of GEMMA (Skretting) Feeds for HPLC Analysis

*GEMMA Micro* 75 and 150, and *GEMMA Wean* 0.2, 0.3, and 0.5 mm pellets were homogenized in a bead beater with 70% methanol. The homogenate was centrifuged, and retained supernatant was dried in a SpeedVac. This was performed by Michelle Price, Ph.D., at Plant Sensory Systems, LLC (Halethorpe, MD, USA). Samples were prepared for HPLC analysis as described in Section 4.8.

### 4.13. Mass Spectrometry

Liver extracts were prepared as described in Section 4.5. 100 μL total liver protein extracts contained 74.7 μg and 52.2 μg from wild-type and sa9430 fish, respectively. These extracts were each incubated with 5 μg rabbit anti-zebrafish CSAD (provided by Plant Sensory Systems, LLC, Halethorpe, MD, USA) with shaking at 1400 rpm for 1 h at 4 °C. 5 μg goat anti-rabbit—biotin (ThermoFisher Scientific, Waltham, MA, USA) was added to each tube, followed by shaking at 1400 rpm for 1 h at 4 °C. 30 μL streptavidin beads from the SMART Digest Immunoaffinity Kit (ThermoFisher Scientific, Waltham, MA, USA) were added followed by shaking at 1400 rpm at 4 °C for one hour followed by an additional hour at 23 °C. The wash protocol outlined in the kit was followed. Digestion occurred at 70 °C for one hour. The supernatant was retained and lyophilized. 

Peptides were analyzed by LC-MS/MS using a Dionex UltiMate 3000 Rapid Separation nanoLC and a linear ion trap—Orbitrap hybrid mass spectrometer (ThermoFisher Scientific, Waltham, MA, USA). Approximately 1 μg of peptide samples was loaded onto the trap column, which was 150 μm × 3 cm in-house packed with 3 um C18 beads. The analytical column was a 75 um × 10.5 cm PicoChip column packed with 1.9 um C18 beads (New Objectives). The flow rate was kept at 300nL/min. Solvent A was 0.1% FA in water and Solvent B was 0.1% FA in ACN. The peptide was separated on a 90-min analytical gradient from 5% ACN/0.1% FA to 40% ACN/0.1% FA. The mass spectrometer was operated in data-dependent mode. The source voltage was 2.10 kV and the capillary temperature was 275 °C. MS^1^ scans were acquired from 400–2000 *m*/*z* at 60,000 resolving power and automatic gain control (AGC) set to 1 × 10^6^. The top ten most abundant precursor ions in each MS^1^ scan were selected for fragmentation. Precursors were selected with an isolation width of 1 Da and fragmented by collision-induced dissociation (CID) at 35% normalized collision energy in the ion trap. Previously selected ions were dynamically excluded from re-selection for 60 s. The MS^2^ AGC was set to 3 × 10^5^. Apex triggering was enabled for the peptide analysis on the Q Exactive HF (ThermoFisher Scientific, Waltham, MA, USA). All setup was same as above except that the top15 most abundant precursor ions in each MS1 scan were selected for fragmentation. Precursors were selected with an isolation width of 2 Da and fragmented by Higher-energy collisional dissociation (HCD) at 30% normalized collision energy in the HCD cell.

Individual raw data files were converted to the vendor neutral mzML format with msconvert [37] and processed with the Trans-Proteomic Pipeline Version 4.8. [38]. Sequence determination was performed using Comet software [39] to search the Swissprot *D. rerio* database, with an abbreviated FASTA containing CSAD sequence 482 amino acids in length (NP_001007349) or the predicted X1 isoform 544 amino acids in length (XP_009295318.1). Methionine oxidation and carbamidomethylation of cysteine were allowed as variable and fixed modifications, respectively. The enzyme specificity parameter was set to trypsin allowing for 1 missed cleavage site per peptide. MS1 precursor ion mass tolerance and MS2 product ion mass tolerance parameters were set to 10 ppm and 0.4 Da, respectively. Peptide spectra matched to theoretical spectra calculated from the database were validated using Peptide Prophet and peptides were assembled into protein groups with Protein Prophet. Sequences were analyzed using MacVector 12.7.5.

## Figures and Tables

**Table 1:** The specific activities of wild-type and sa9430 CSAD enzymes are statistically indistinguishable. Rates were calculated based on conversion of cysteic acid to taurine. CSAD activity assays were performed as described in Materials and Methods.

Strain	pmol Taurine/h·μg Protein
Wild-type	5.83 ± 1.94
sa9430	3.09 ± 0.45
